# Medical Image Classification Based on Semi-Supervised Generative Adversarial Network and Pseudo-Labelling

**DOI:** 10.3390/s22249967

**Published:** 2022-12-17

**Authors:** Kun Liu, Xiaolin Ning, Sidong Liu

**Affiliations:** 1 School of Information Engineering, Shanghai Maritime University, Shanghai 200135, China; 2 Australia Institute of Health Innovation, Macquarie University, Sydney 2113, Australia

**Keywords:** digital histopathology, deep learning, generative adversarial network, *k*-means clustering, medical images classification, semi-supervised learning

## Abstract

Deep learning has substantially improved the state-of-the-art in object detection and image classification. Deep learning usually requires large-scale labelled datasets to train the models; however, due to the restrictions in medical data sharing and accessibility and the expensive labelling cost, the application of deep learning in medical image classification has been dramatically hindered. In this study, we propose a novel method that leverages semi-supervised adversarial learning and pseudo-labelling to incorporate the unlabelled images in model learning. We validate the proposed method on two public databases, including ChestX-ray14 for lung disease classification and BreakHis for breast cancer histopathological image diagnosis. The results show that our method achieved highly effective performance with an accuracy of 93.15% while using only 30% of the labelled samples, which is comparable to the state-of-the-art accuracy for chest X-ray classification; it also outperformed the current methods in multi-class breast cancer histopathological image classification with a high accuracy of 96.87%.

## 1. Introduction

The design and use of artificial intelligence (AI), especially deep learning (DL), is driving fundamental changes in natural language processing, visual object recognition and many other domains [1]. Since AlexNet [2] won the ImageNet Challenge in 2012, DL models have dramatically improved the state-of-the-art in object detection and image classification at large scale [3]. DL also holds promises in transforming healthcare and medicine, with encouraging results recently reported in skin cancer classification [4], pneumonia detection [5], glioma prognosis [6], diabetic retinopathy detection [7], glaucoma screening [8], interstitial lung diseases classification [9], and most recently, COVID-19 assessment [10,11], etc. DL models usually require a large number of labelled samples to train; therefore, great effort has been taken to collect and label large-scale datasets, such as the ImageNet [3] and the Winograd Schema Challenge [12], and many researchers are motivated to participate in public computational challenges to take advantage of such datasets. However, it is challenging to acquire large-scale medical image datasets, as medical images usually have restricted accessibility and require clinical expertise to annotate. These limitations hamper the translation of DL models to medical image classification.

To reduce the dependence on large-scale expert-annotated medical image datasets, several unsupervised learning methods were proposed. Deep Embedding for Clustering (DEC) [13] is one of the first unsupervised methods to cluster unlabelled data, which is based on self-training. CosFace [14] used the estimated clustering uncertainty of unlabelled samples to adjust the loss function weight to reduce the overlapping-identity label noise; however, it requires balanced labelled and unlabelled samples to estimate clustering uncertainty accurately, which is a major limitation. There are a few methods based on transfer learning [15,16] and meta-learning [17,18]. Ahn et al. [15] proposed a hierarchical unsupervised feature extractor, which has a convolutional autoencoder on top of a pre-trained convolutional neural network (CNN). Arti Pet al. [16] fine-tuned the pre-trained AlexNet [2] and GoogleNet [19] for X-ray image classification. Maicas et al. [18] designed an unsupervised pretext task for meta-learning and then trained the model for medical image classification. However, due to the lack of domain experts’ input, it is difficult for these unsupervised methods to meet the high sensitivity and specificity requirements for medical applications.

The past few years have seen an emerging application of semi-supervised learning in many computer vision tasks. Semi-supervised learning methods usually require fewer expert-annotated samples (less labelling cost) and can also take advantage of a large amount of unlabelled data (more training data). In a recent survey, Van Engelen and Hoos [20] provided an overview of semi-supervised learning methods, most of which were based on a Generative Adversarial Network (GAN) [21]. GAN is a very successful unsupervised learning method for data synthesis with a wide range of applications in medical image computing [22], such as color normalisation [23], and has been used to overcome the problem of insufficiently labelled data. Odena et al. [24] developed a class-conditional GAN for image synthesis to augment training data. GAN models are usually difficult to train with known issues like mode collapse and failure to converge; therefore, variant GAN models were proposed with improved reliability. Han et al. [25] proposed a conditional GAN based on Bayesian uncertainty estimation and noise-tolerant adversarial learning, which was validated on datasets with low dimensionality demonstrating robust performance in noise resistance. Guo et al. [26] proposed a positive-unlabelled GAN (PU-GAN), which divided the generated images into positive or negative samples based on image quality to reduce the high heterogeneity in sample quality. These GAN models improved the stability and quality of the generated samples and achieved better performance than those sophisticated discriminator stabilisation methods.

Semi-supervised learning has also been used in medical image classification. In one of our recent studies, we developed a semi-supervised GAN (SSGAN) for lung X-ray classification, which only requires a small number of labelled samples [27]. This model extended the unsupervised GAN by adding an additional class of GAN-synthesised images to guide the training process. SSGAN is able to estimate the distribution of both labelled and unlabelled data so that the discriminator network, i.e., the classifier, is more robust than those trained on the labelled samples alone. We believe SSGAN can be further improved by integrating with pseudo-labelling, i.e., assigning pseudo labels to unlabelled samples based on their distances to the labelled sample cluster centres. In this study, an enhanced semi-supervised GAN with pseudo-labelling (PLAB-GAN) is proposed for medical image classification, which can not only use unlabelled data to estimate the sample distribution but also train the classifier directly.

In summary, in this study, we have made the following contributions.

- A novel GAN model based on pseudo-labelling and semi-supervised learning was proposed to optimise the use of unlabelled data in medical image classification.
- The proposed method is methodologically innovative. We used ResNet-20 to extract features from unlabelled data and further inferred their labels based on K-means clustering. We also customised the discriminator network of GAN by converting it to a multi-class classifier, which is not only able to classify if a sample is real or fake but also to predict its class. These methods effectively strengthened the effect of image features on classification, alleviated the problem of the unobvious intra-class gap and improved the accuracy of pseudo-labelling.
- We conducted extensive experiments on two benchmark datasets, including ChestX-ray14 [28] and BreakHis [29], and demonstrated that our method could improve the state-of-the-art performance of medical image classification for lung disease diagnosis using an X-ray and for breast cancer diagnosis using histopathology images.

## 2. Materials and Methods

**Figure 1:**
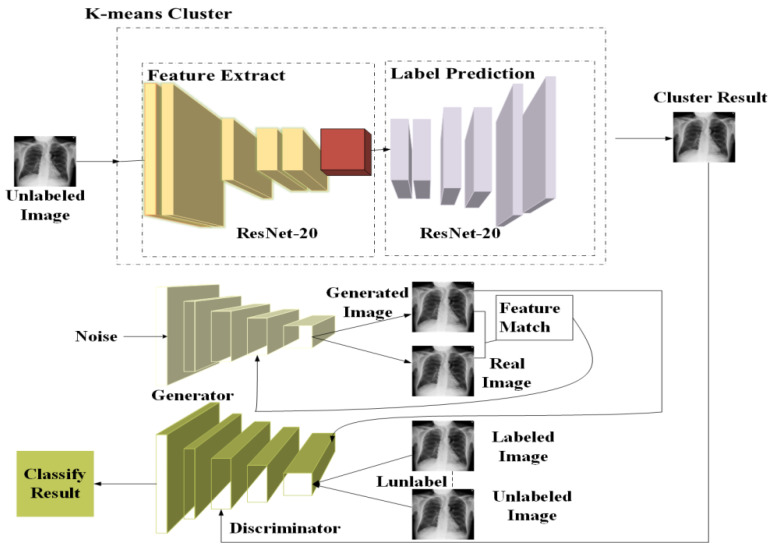
Overiew of the proposed pseudo-labelling-based semi-supervised generative adversarial network (PLAB-GAN).

A novel medical image classification method was proposed by integrating pseudo-labelling into semi-supervised GAN (PLAB-GAN). The overall framework of PLAB-GAN is illustrated in Figure 1. We first clustered the unlabelled samples to the cluster centres of the labelled images to estimate pseudo labels based on the CNN features extracted from the samples using a pretrained ResNet-20 network. Secondly, from each cluster, a small number of labelled data (X_lab) and a greater number of unlabelled data (X_unlab) were selected to train the discriminator/classifier, which classifies the samples into K classes (the number of classes of the real data). We further added a new class to the discriminator output for the synthetic data (X_gen) so that the synthetic images can be classified into the K+1 category (K classes for the real data and 1 pseudo class for the synthetic data). While training the PLAB-GAN, the discriminator and generator networks were alternately updated until reaching a certain number of iterations. Finally, the trained discriminator was used for medical image classification.

### 2.1. Pseudo-Labelling Based on K-Means Clustering

Pseudo labels, which are the estimated labels of unknown data, are generally used for processing large-scale unlabelled data. In this study, we chose K-means clustering to estimate the pseudo labels of the unlabelled images for their simplicity and robustness. Size (128 × 128 pixels) and intensity [−1,1] normalisation were first applied to the images. The preprocessed labelled images were then used to train a RestNet-20 which was pre-trained on ImageNet [3]. The network was trained through iterative learning as:

(1) $$\mathrm{label}=\mathrm{argmin}\{\overset{K}{\underset{i=1}{\sum }}w^{i}{\left(x^{i}-x_{1}^{i}\right)}^{2},\overset{K}{\underset{i=1}{\sum }}w^{i}{\left(x^{i}-x_{2}^{i}\right)}^{2}\ldots \ldots \overset{K}{\underset{i=1}{\sum }}w^{i}{\left(x^{i}-x_{j}^{i}\right)}^{2}\}$$

where $x^{i}$ indicates the average feature vector, i.e., cluster centre, of the *i*th class ($i=\left\{1,\ldots ,K\right\}$), $x_{j}^{i}$ represents the feature vector of each image, and $w^{i}$ express features weight. The output of activation function, *p*, which is the probability that the sample *x* belongs to each class, was then assigned to $w^{i}$ to update the network weights. The last layer used Softmas as activation function, and the other layers all used ReLU.

The trained network is then used to extract features from the unlabelled samples for subsequent clustering and pseudo-labelling. For each unlabelled image, x_unlab, the Euclidean distance between its feature f(x_unlab) and each cluster centre $x_{i}$ was used to estimate the pseudo label.

### 2.2. Generative Adversarial Network

A generated adversarial network (GAN) consists of a generator and a discriminator. GAN uses the idea of confrontation training which is based on game theory. A generator network G aims to produce images (x) by transforming vectors of noise z (x = G(z)) that are similar to the real images. The discriminator network D is trained to distinguish data generated from the generator distribution $p_{z}$ from real data. The generator network, in turn, is then trained to fool the discriminator into accepting its outputs as being real. During GAN model training, the generator G(z) and the discriminator D(x) will update their own parameters to minimise the loss. Through continuous iterative optimisation, a Nash equilibrium state—the optimal state—is finally reached by the two networks. The objective function of discriminator is defined as:

(2) $$s^{D}=-E_{x-p_{data}}lb\left[D\left(x\right)\right]-E_{z-p_{z}}lb\left\{1-D\left[G\left(z\right)\right]\right\}$$

and the objective function of the generator is defined as:

(3) $$s^{G}=-E_{z-p_{z}}lbD\left[G\left(z\right)\right]$$

where *D*(*) is the discriminant probability of the discriminator, *G*(*z*) is the generated image, lb represents the logarithm with a base of 2, $z-p_{z}$ indicates the noise with random distribution, $x-p_{data}$ is the image data that follows random distribution.

### 2.3. Classification Based on GAN

The classification model we used was based on a previously proposed semi-supervised GAN (SSGAN) [27]. For a *K*-class classification problem, we added a new class to the discriminator Softmax output for the synthetic data, i.e., K classes for the real data and 1 class for synthetic data. Pseudo labels of the unlabelled images were inferred from the cluster centres of labelled samples, which were generated from K-means clustering.

The noise vectors following a normal distribution of (0, 1) were fed into the generator to generate synthetic images. The input noise vector was first converted into a one-dimensional vector by a fully connected layer (dense) and then reshaped to dimensions of 32 × 32 × 256. Following the dense layer are two blocks of layers, each consisting of a 2D deconvolution layer (stride of 2 pixels), a batch norm layer, and an activation layer. For the activation function, the final output layer uses Tanh activation function, and the rest of the layers use LeakyReLU (slope on the negative half-axis was set to 0.01). Compared with the ReLU activation function, this activation function adds a linear correction unit to deal with negative input values. The size of the final generated image is 128 × 128 × 1.

The generated images, a small number of labelled images and a larger number of unlabelled images were then used to train the discriminator. The discriminator network includes a conv2d layer, an activation layer (output dimensions: 64 × 64 × 32), a 2nd conv2d layer, a batch norm layer, an activation layer (output dimensions: 32 × 32 × 64), a 3rd conv2d layer, another batch norm layer and activation layer (output dimensions: 16 × 16 × 128). The convolution layers take stride of 2 pixels with convolutional kernels of 3 × 3. The activation function uses LeakyReLU, with the slope on the negative half-axis set to 0.01. The last three layers of the discriminator network are a flattened layer (to convert tensor to one dimension vector), a dropout layer (to prevent overfitting) and a dense layer.

### 2.4. Loss Functions

The output of the discriminator was a K + one-dimensional logical vector, $\left\{l_{1},l_{2}\ldots l_{k+1}\right\}$ which was calculated by Softmax. The first K elements of the vector ($l_{1},l_{2}\ldots l_{k}$) represent the probabilities of being the real classes, $l_{k+1}$ represents the probability of being the synthetic class. The probability of a sample (*x*) being a specific class (*i*) can be calculated as:

(4) $$p\left(y=i|x\right)=\frac{\exp\left(l_{i}\right)}{\sum_{j=1}^{k+1}\exp\left(l_{j}\right)}$$

where $\overset{k+1}{\underset{j=1}{\sum }}\exp\left(l_{j}\right)$ represents the sum of the probability values over the K+1 classes

The categorical cross-entropy loss was used for the labelled image classification. Binary cross-entropy was used for unlabelled images and generated images, i.e., probabilities of the sample belonging to a real class or a synthetic class. There are three types of images in the discriminator: generated images, labelled images, and unlabelled images; therefore, three types of loss functions are designed, as in Equations (5)–(7):

(5) $$l_{label}=-E_{\left(x,y\right)-p_{data}}\left[lnp\left(y|x\right),y<k+1\right)]$$

(6) $$l_{unlabel}=-E_{x-p_{data}}\left[\ln\left(1-p\left(y=k+1|x\right)\right),y=k+1\right)]$$

(7) $$l_{gen}=-E_{x-G}\left[lnp\left(y=k+1|x\right),y=k+1\right)]$$

where *x* represents the image, *y* represents the label of the image, *x*-$p_{data}$ represents the image without label and *x*-*G* represents the generated image, (*x*,*y*)-$p_{data}$ represents the image with label, *p*(|) indicates the predicted probability, $l_{label}$ is the cross-entropy loss of the true and the predicted class label distributions for the labelled samples, $l_{unlabel}$ is the loss for the unlabelled samples classified as a true class, and $l_{gen}$ is the loss for generated samples classified as real samples. The loss function of the discriminator ($l_{d})$ is the sum of $l_{label}$, $l_{unlabel}$ and $l_{gen}$, as in Equation (8), where α and β represent the weight on $l_{unlabel}$ and $l_{gen}$, respectively.

(8) $$l_{d}=l_{label}+\alpha l_{unlabel}+\beta l_{gen}$$

The discriminator D and the generator G were trained alternatively. When training D, the weights of G were fixed, and Adam method was used to update the weights of D. Then, the weights of G were optimised by matching the features between the real and the generated images. The above steps were repeated until there was no further improvement of the model or the maximum number of iterations (*n* = 15,000 for ChestX-ray14 and *n* = 150 BreakHis) was reached.

## 3. Results

We tested the proposed method on two benchmark datasets, including the ChestX-ray14 dataset [28] and BreakHis [29]. All experiments were performed on a workstation with a 16GB GPU (NVIDIA, GeForce GTX1080TI). The algorithm was implemented in Python 3.6. To verify the results, we repeated the experiments 10 times and the mean accuracy values were reported. The datasets, experiments and results are described below.

### 3.1. Chest X-ray Pseudo-Labelling Results

**Figure 2:**
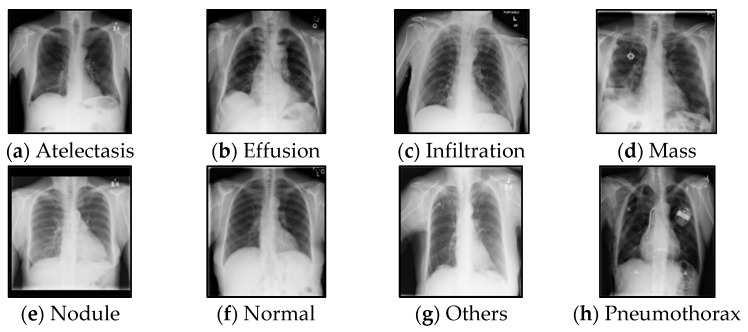
The chest X-ray sample images.

The ChestX-ray14 dataset contains 112,120 chest X-ray images labelled with 14 types of lung diseases. We selected seven types of common diseases and a normal control class for chest X-ray classification, as shown in Figure 2. In the *K*-means clustering, we used 16,089 labelled samples (~2100 from each class, with a train:test ratio of 7:3) to train the ResNet-20 network, which was later used to extract features from unlabelled X-ray images. Then *K*-means clustering was used to infer the pseudo labels of 8583 unlabelled samples. To train the semi-supervised GAN for classification, we used a small number of labelled images (n = 50 to 400 per class) and a large number of unlabelled images (n = 8583). The X-ray dataset was divided into training:test:validation split was set to 7:2:1. The GAN learning rate is 0.0001 and the batch size is set to 16. The semi-supervised experiment uses Adam to optimise the loss function with a momentum of 0.5. We used accuracy as the metric to validate the effectiveness of the method, as in Equation (9), where TP is True positive, FN is False Negative, TN is True Negative, and FP is False Positive.

(9) $$\mathrm{accuracy}=\frac{\mathrm{TP}+\mathrm{TN}}{\mathrm{TP}+\mathrm{TN}+\mathrm{FP}+\mathrm{FN}}$$

**Figure 3:**
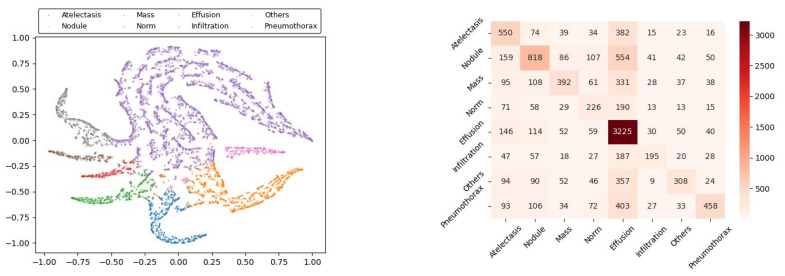
The visual representation of K-means clustering results: feature distribution after clustering (**left**); distances between the predicted label and the real label (**right**).

**Figure 4:**
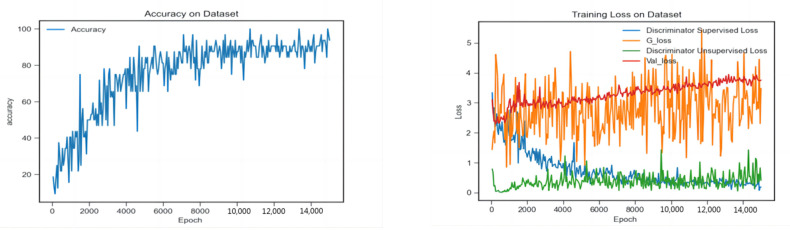
The model’s performance, accuracy (**left**) and training loss (**right**) on the X-ray dataset.

Figure 3 shows the visual representations of K-means clustering results. Figure 4 shows the experimental results using 400 labelled images in each class: changes in the accuracy (left) and the loss (right) of the model during the training process. It can be seen that the classification accuracy reached 0.860 ± 0.026 after 8000 epochs and further improved to 0.930 ± 0.032 after 15,000 epochs. The discriminator loss decreased continuously, whereas the generator loss quickly decreased in the early stage and then increased slightly later. The discriminator loss was lower than the generator loss, indicating the discriminator could distinguish the generated images very well.

### 3.2. Chest X-ray Classification Results

To verify the effectiveness of the proposed method, we compared it with convolutional neural network (CNN), PCA-based semi-supervised method (PCA + SVM) and GAN-based semi-supervised method (SSGAN). To investigate the impact of the amount of the labelled samples for training, the experiments were repeated five times with different settings in terms of the number of images per class for training, i.e., 50, 100, 200, 300 and 400, respectively. Table 1 shows the classification accuracy with different numbers of labelled images using different networks. Compared to CNN, PCA + SVM and SSGAN, the proposed method achieved substantially better performance. Increasing the number of labelled training images improves all the models’ performance. The largest performance gain was seen when using 400 labelled samples per class for training. The proposed method outperformed CNN, PCA + SVM and SSGAN by 18%, 20% and 16%, respectively.

Table 2 shows the classification accuracy (with 400 labelled training images per class) of CNN, SSGAN and the proposed method in individual classes. The proposed method outperformed SSGAN and CNN in five out of six classes, except for the Mass class.

**Figure 5:**
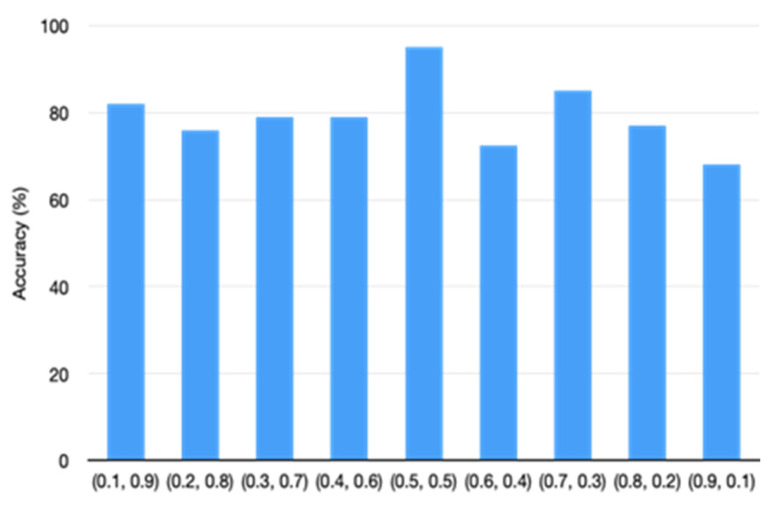
Classification accuracy with different $\left(\alpha ,\beta \right)$ values.

To investigate the impact of loss weight parameters α and β on the model’s performance, we tested different parameter settings (values ranging from 0.1 to 0.9). Figure 5 shows the corresponding classification accuracy in different settings. It shows that when α and β both were equal to 0.5, the model achieved the highest classification accuracy.

### 3.3. BreaKHis Pseudo-Labelling Results

**Figure 6:**
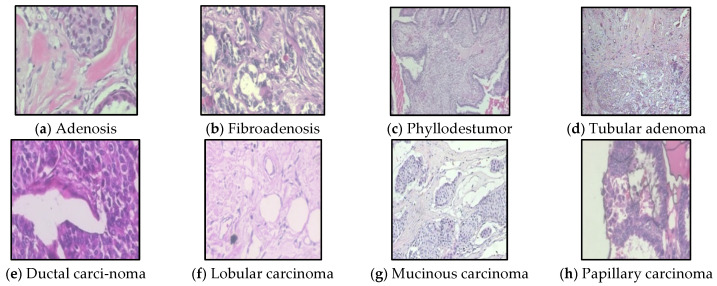
Breast cancer histopathology images from the BreakHis dataset.

We tested the proposed method on a second benchmark dataset–BreaKHis [29], which contains 7909 breast tissue microscopic images, including 2480 benign and 5429 malignant samples across eight sub-types (benign subtypes: adenosis, fibroadenoma, phyllodes tumor, and tubular adenoma; malignant subtypes: ductal carcinoma, lobular carcinoma, mucinous carcinoma, and papillary carcinoma), as shown in Figure 6. The images were acquired using an Olympus BX-50 system microscope and a relay lens with a magnification of 3.3× attached to a Samsung digital color camera SCC-131AN. The images were in the three-channel RGB true color space (8 bits per channel) with different magnifications (40×, 100×, 200×, 400×). In the experiment, we expanded the dataset to 14,523 through rotation and translation operations.

To run K-means clustering, we selected 3191 labelled breast cancer images (~400 samples per class) to train the ResNet-20 network with a train:test ratio of 7:3. Image features were then extracted by the trained ResNet-20 for subsequent K-means clustering and pseudo-labelling of a total of 10,162 unlabelled breast cancer images. The same network structure, parameters and evaluation metrics as for the X-ray classification experiment were used in this experiment.

**Figure 7:**
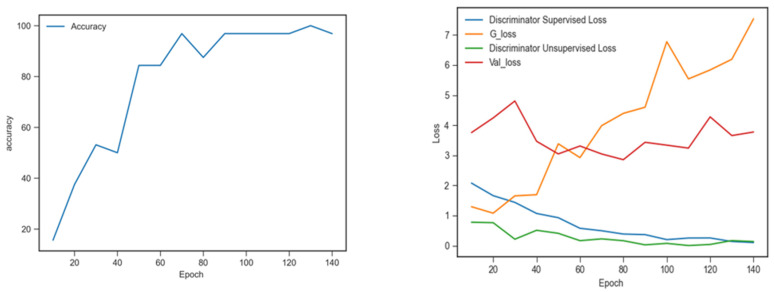
Classification performance on BreakHis dataset: accuracy (**left**), training loss (**right**).

Figure 7 shows the classification performance on the BreakHis dataset. It shows that the classification accuracy was the highest (0.9687) after 140 epochs. From Figure 7 (right), it can be seen that the discriminator loss gradually decreased during the training; the discriminator loss was lower than the loss of the generator, indicating that the discriminator performed well in recognition of the labelled, unlabelled and synthetic samples.

### 3.4. BreaKHis Classification Results

For the breast cancer image classification experiment, three different settings in terms of the number of labelled training samples were tested, i.e., 10, 20 and 30, respectively. Table 3 shows the accuracy of the model compared to CNN and SSGAN. We also compared our results with the recently published results on the same dataset, including ResNet50 [30,31] and three other types of CNN/DNN models [32,33,34,35]. The comparison with these methods is shown in Table 4. Table 5 further shows the classification accuracy in each individual subtype. As can be seen from Table 3, Table 4 and Table 5 the proposed algorithm achieved very good overall classification performance (96.87%) and also consistently high performance (95.60–97.31%) across different subtypes while using only a small number of labelled samples. It also outperformed the state-of-the-art methods.

## 4. Discussion

A bottleneck exists in supervised learning for medical image classification. It is difficult to obtain a large number of labelled medical images for training due to the restriction in accessing, sharing and labelling patients’ data. Developing robust and effective DL models with limited labelled data remains a major challenge in computer vision tasks, including medical image classification. To address this challenge, we proposed a novel method that used pseudo-labelling and semi-supervised GAN to classify medical images. This method effectively reduced the dependence of DL models on large-scale labelled data. The technical innovation of our method firstly includes our training of a ResNet-20 model to extract image features from the medical images, which could robustly assign pseudo labels to unlabelled images; secondly, our method enforcing the similarity between similar image features and minimising the intra-class distances, which strengthened pseudo-labelling performance and also improved the characterisation of image features effectively.

While DL will be increasingly used in medical image classification, how to use a large amount of information in unlabelled medical images is an emerging research area. Our proposed method provides a feasible solution for medical image classification, which uses a small amount of labelled data but can achieve equivalent or better performance compared to supervised learning methods. Our study demonstrates that pseudo-labelling and semi-supervised GAN might be a good option for the future development of intelligent medical image classification systems.

One limitation of the proposed method is that the quality of the ground truth labels has a huge impact on the clustering/pseudo-labelling, as well as the subsequent classification performance. If the ground truth labels are incorrect, the pseudo labels based on them will become less reliable; thus, the errors will propagate to the feature extractor and classifier, leading to misclassification and lower classification accuracy. Potential solutions to this problem include a mechanism to detect out-of-distribution samples [36], such as anomalies and adversarial samples, from the training set and novel ambiguity quantification functions [37] to regulate the weights of unreliable training samples. This will be investigated in our future studies.

## 5. Conclusions

To improve the classification accuracy of medical images and reduce the use of labelled images, we proposed a novel method based on *K*-means clustering/pseudo-labelling and semi-supervised GAN. Comprehensive experiments were carried out on two benchmark datasets, including ChestX-ray14 and BreakHis. The results demonstrate that our algorithm outperformed the state-of-the-art methods and worked effectively in medical image classification with a small number of labelled samples. It achieved 93.15% accuracy in X-ray classification with 400 labelled images per class and 96.87% accuracy in breast histopathology image classification with only 30 labelled images per class. The method has a high potential to assist in tasks where the unlabelled data is rich, but the labelling cost is high. In our future studies, we will further investigate novel strategies to enhance the model’s performance and robustness.

## Figures and Tables

**Table 1:** Classification accuracy of samples with different numbers of labelled data.

No. of Labelled Samples in Each Class	Accuracy (%)			
	PCA + SVM	CNN	SSGAN	The Proposed
50	58.94 ± 6.3	55.62 ± 6.2	62.47 ± 4.7	**70.60 ± 5.0**
100	63.20 ± 4.1	61.64 ± 5.5	68.71 ± 4.3	**73.84 ± 3.2**
200	67.76 ± 4.6	68.89 ± 2.0	72.40 ± 4.0	**78.69 ± 4.8**
300	68.60 ± 5.6	72.85 ± 5.0	74.25 ± 3.5	**84.92 ± 2.6**
400	70.10 ± 3.9	74.54 ± 3.4	77.83 ± 3.8	**93.15 ± 3.2**

* Bold type represents the best result.

**Table 2:** Classification accuracy of different models for individual classes.

Class	Accuracy (%)		
	CNN	SSGAN	The Proposed
Atelectasis	79.4	81.97	**94.0**
Nodule	75.2	77.38	**96.0**
Mass	**88.4**	85.32	84.0
Effusion	88.2	82.75	**91.0**
Infiltration	70.5	71.62	**86.0**
Pneumothorax	87.8	83.41	**93.0**

* Bold type represents the best result.

**Table 3:** Classification accuracy of samples with different numbers of labelled data.

Labelled Data	Accuracy (%)		
	CNN	SSGAN	The Proposed
10	68.87	75.37	95.10 ± 0.20
20	72.35	81.36	96.00 ± 0.70
30	73.68	84.63	96.87 ± 0.50

**Table 4:** Classification accuracy of different models.

Model	Accuracy (%)
Y. Yari et al. [30]	93.35
M. Nawaz et al. [31]	95.00
P. Nguyen et al. [32]	73.68
S. Pratiher et al. [33]	95.46
D. Bardou et al. [34]	88.23
Z. Han et al. [35]	93.80
The Proposed	96.87

**Table 5:** The classification accuracy of each type of breast cancer disease data.

Major Class	Subclass	Accuracy (%)
benign	adenosis	96.12
	fibroadenoma	96.88
	phyllodes tumor	96.74
	tubular adenoma	95.60
malignant	ductal carcinoma	97.31
	lobular carcinoma	96.80
	mucinous carcinoma	95.78
	papillary carcinoma	96.87

## Data Availability

Two public benchmark datasets were used in this study, including the ChestX-ray14 dataset [28] and BreakHis [29].
